# Enduring Legacy: Tracing the History of the Berlin Summer School on “Psychiatry as a Science”

**DOI:** 10.1007/s40596-024-02018-1

**Published:** 2024-08-05

**Authors:** Shuyan Liu, Andreas Heinz, Andreas Ströhle, Norman Sartorius, Hanfried Helmchen

**Affiliations:** 1 Charité – Universitätsmedizin Berlin, Berlin, Germany; 2 German Center for Mental Health (DZPG), Partner Sites Berlin/Potsdam, Germany; 3 Association for the Improvement of Mental Health Programmes, Geneva, Switzerland

To the Editor:

In August 2000, against the backdrop of recovery from wars in Eastern Europe, the inaugural Berlin Summer School on “Psychiatry as a Science” emerged as a beacon of hope and a catalyst for positive transformation in the regions.

Following the Balkan war in the 1990s, Professor Dieter Simon, the President of the Berlin-Brandenburg Academy of Sciences and Humanities (BBAW), proposed supporting scientific (re)development in the Balkan countries. Professor Hanfried Helmchen, as a member of the BBAW, in collaboration with Professor Norman Sartorius devised a plan for a summer school. It was aimed to bring together young psychiatrists from European countries affected by the wars in the former Yugoslavia to foster their mutual recognition and collaboration and enhance psychiatric education and research in the region facing post-conflict challenges. To bring this vision to fruition, they sought funding from the Volkswagen Foundation. Since 2002, Professors Andreas Heinz and Andreas Ströhle have undertaken the organization of the summer school, which took place annually at the BBAW for its first decade and then moved to Charité starting in 2010.

Once the financial support from the Volkswagen Foundation was received, psychiatrists from countries which made up Yugoslavia and some other Eastern European countries were invited to apply. One or two applicants from each of these countries were selected, totaling 15 participants who attended the first Berlin Summer School on “Psychiatry as a Science” August 26–30, 2000. Over the course of an intense week, participants were deeply engaged in a dynamic learning environment, making it possible to acquire leadership and professional skills, make presentations, participate in discussions led by esteemed experts, and visit research labs and clinical wards. From dawn till dusk, participants delved into a variety of topics ranging from research skills to clinical practice. Participants were assigned various tasks, including introducing their workplace and each other, delivering a presentation, and presenting a poster. Their presentations were critiqued and refined by the entire group, both for content and the presentations’ techniques, fostering a culture of constructive feedback and continuous improvement. Throughout its courses, the Berlin Summer School prioritized the development of skills essential for personal and professional development, such as refining presentation techniques, writing a compelling letter of motivation, polishing a curriculum vitae, addressing a question, reading and writing academic papers, formulating titles, chairing meetings, presenting oneself for elections, and prioritizing tasks. By the end of the week, participants provided valuable and constructive feedback, which was used in developing plans for future work. It was evident that participants had never experienced such intensive training and learned so much.

It can be asserted that the primary aim of the school was achieved because participants from the countries affected by recent wars came together, collaborated during the program, and continued their collaboration afterward. The Berlin Summer School is believed to be the inaugural scientific event that brought together young psychiatrists from nations previously in conflict.

Over time, the courses brought together participants from Eastern and Western Europe, including Germany. The program attracted attendees from Albania, Azerbaijan, Belarus, Bosnia and Herzegovina, Bulgaria, China, Croatia, the Czech Republic, Estonia, Germany, Hungary, Iran, Ireland, Israel, Italy, Latvia, Lithuania, Macedonia, Moldova, Montenegro, Mexico, Poland, Portugal, Romania, Russia, Serbia, Slovakia, Slovenia, Spain, Taiwan, Turkey, Ukraine, and the USA.

An important feature of the Berlin Summer School was its dedication to nurturing research innovation among participants. Starting from the third Berlin Summer School, in 2002, participants were encouraged to draft and present their research proposals with other participants in the group. This initiative sparked collaborative projects among participants, fueling creativity in the years to come. As a result, these endeavors were not only intellectually fulfilling but also gained international recognition. Two out of three research proposals obtained third-party funding. Moreover, several findings were published in international journals [1–4]. One of the international collaborative studies carried out by the participants received the prestigious Okasha Prize from the World Association of Psychiatry for its groundbreaking insights into the pathways to psychiatric care in Eastern Europe [1]. In addition, the Berlin Summer School was acknowledged by the Volkswagen Foundation as a pioneering role model of effective investment in research and professional advancement.

Beyond the immediate impact of the Berlin Summer School, its legacy endured through the establishment of the East European Psychiatry Initiative (EEPSI). This research network provided a platform for research and professional advancement, facilitating collaboration and knowledge exchange.

The Berlin Summer School continued every year, except during the COVID-19 pandemic (2020–2021). Over the past decade, its scope expanded from Eastern Europe to a global scale by inviting junior psychiatrists worldwide. The global outreach facilitated cross-cultural understanding of mental health and highlighted the unmet need for collaboration among countries and within countries to facilitate the provision of mental health care in settings differing in the availability of resources, culture, and organization of services. The twentieth Summer School took place in 2023, in collaboration with the German Center for Mental Health (DZPG) and the Association for the Improvement of Mental Health Programmes. Notably, it featured the participation of three junior psychiatrists from Ukraine.

The summer school stands as a testament to driving transformative change in mental health education and practice, capacity-building, and research innovation [5]. Its enduring legacy continues to resonate through the achievements of its alumni, inspiring and empowering future generations of mental health experts to their journey toward mental health care and research, disseminating research findings, and shaping the future of mental health in Eastern Europe and beyond.
